# Design and Analysis of Tubular Permanent Magnet Linear Wave Generator

**DOI:** 10.1155/2014/258109

**Published:** 2014-06-23

**Authors:** Jikai Si, Haichao Feng, Peng Su, Lufeng Zhang

**Affiliations:** School of Electrical Engineering and Automation, Henan Polytechnic University, Jiaozuo 454000, China

## Abstract

Due to the lack of mature design program for the tubular permanent magnet linear wave generator (TPMLWG) and poor sinusoidal characteristics of the air gap flux density for the traditional surface-mounted TPMLWG, a design method and a new secondary structure of TPMLWG are proposed. An equivalent mathematical model of TPMLWG is established to adopt the transformation relationship between the linear velocity of permanent magnet rotary generator and the operating speed of TPMLWG, to determine the structure parameters of the TPMLWG. The new secondary structure of the TPMLWG contains surface-mounted permanent magnets and the interior permanent magnets, which form a series-parallel hybrid magnetic circuit, and their reasonable structure parameters are designed to get the optimum pole-arc coefficient. The electromagnetic field and temperature field of TPMLWG are analyzed using finite element method. It can be included that the sinusoidal characteristics of air gap flux density of the new secondary structure TPMLWG are improved, the cogging force as well as mechanical vibration is reduced in the process of operation, and the stable temperature rise of generator meets the design requirements when adopting the new secondary structure of the TPMLWG.

## 1. Introduction

As coal, oil, natural gas, and other nonrenewable energy sources are drying up, all countries in the world are promoting the development process of clean renewable wave energy. The permanent magnet linear wave generator which can convert wave energy directly and efficiently has got the attention of scholars.

A permanent magnet linear generator structure with nine skewed mounted permanent magnets on the mover was proposed in [1, 2]. Reference [3] presented a multiphase tubular linear wave generator which uses laminated permanent magnets structure to form invalid poles on the mover. In [4, 5], a symmetry linear generator whose stator coil uses no vertical overlap structure is presented. References [6–8] and [6] analyzed a new type of switch magnetic flux permanent magnet linear generator structure. References [9, 10] show stator teeth which are semicircular arc and the permanent magnet is inserted in the middle of mover and the coil windings are embedded in the side slot of mover. Reference [11] puts forward a novel permanent magnet tubular linear generator buoy system for converting the ocean waves into electrical energy. In the novel technology for the design of TPMLWG, such as a seawater air gap in the generator between the magnet and armature, cogging force reduction techniques and radially oriented laminations were proposed. In [4], a short primary and long secondary permanent magnet linear generator was designed to reduce the amount of copper. Reference [12] described the design criteria of a tubular linear motor with interior permanent magnets. Considering the effect of the motor dimensions and PM materials, force-to-volume ratio of different motor design solutions has been compared. In [13], tubular linear permanent magnet motors, including different topologies, that is, interior and surface-mounted PMs, and slotted and slotless motors have been comprised of analysis, using preliminary analytical considerations and the further 2D and 3D finite-element analysis. However, there are some problems in existent permanent magnet motor such as lacking mature design program, poor sinusoidal characteristics of air gap flux density, big magnetic flux leakage, and low efficiency.

Keeping in mind the above considerations, a design method and a new secondary structure of the TPMLWG are proposed in this paper, which have satisfactory stable temperature rise and meanwhile improve the sinusoidal characteristics of air gap flux density as well as reducing the cogging force and mechanical vibration in the operating process of the TPMLWG.

## 2. Structure Design

### 2.1. Basic Structure of TPMLWG

**Figure 1:**
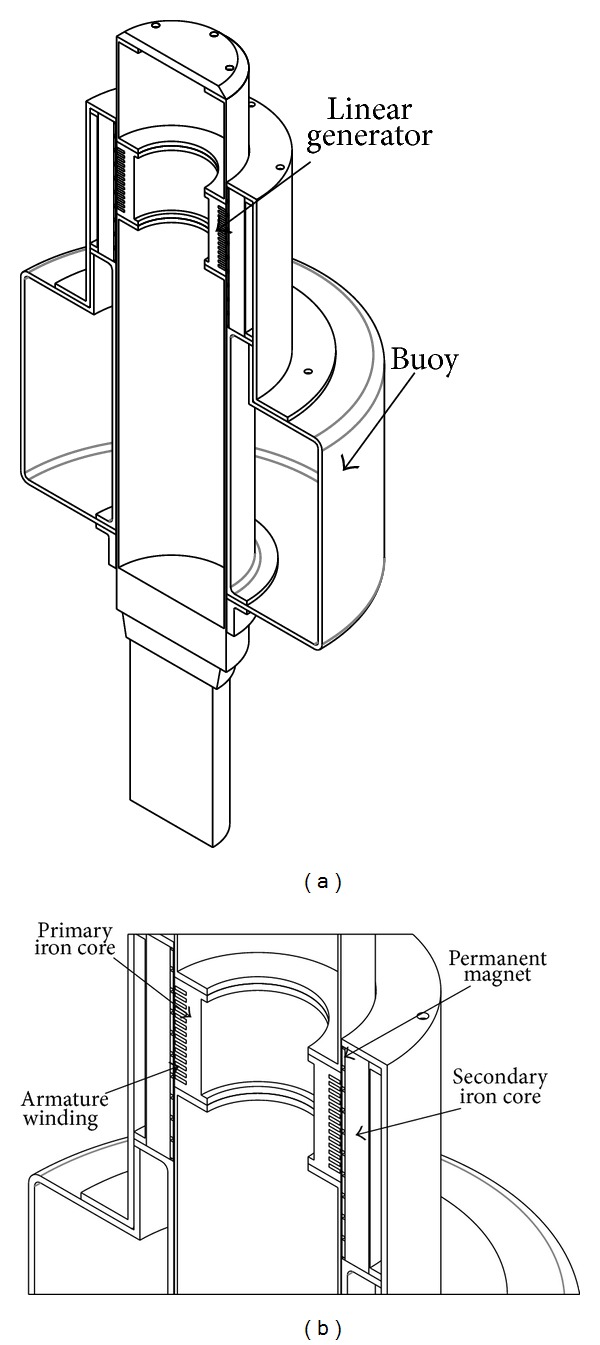
Structure of TPMLWG: (a) the whole structure of TPMLWG and (b) the structure of linear generator.

TPMLWG consists of primary part and secondary part. The primary part is made up of primary iron core, armature windings, and central axis. The armature windings which are fixed with epoxy resin or slot wedge are embedded in the primary iron core slot in a certain way, and the primary iron core is located in the middle of the central axis which is divided into two parts. The secondary part, which consists of permanent magnet, secondary iron core, and buoy, does relative movement as waves, generates magnetic field lines, and induces electromotive force in the armature windings [11]. The basic structure is shown in Figure 1.

In the paper, TPMLWG is designed with long secondary and short primary part to reduce the amount of copper in the armature windings, so as to decrease the copper loss and improve the efficiency of the generator. The primary part is placed in the center, and the rabbets face the secondary part in order to embed armature windings conveniently and simplify the manufacture technology of the primary part effectively. For the basic structure of TPMLWG shown in Figure 1, it is the same as the structure in [11], but a new secondary structure, which can improve the air gap flux density of TPMLWG, is proposed in this paper.

### 2.2. Design Method of TPMLWG

For the design method of TPMLWG, the main dimensions are determined preliminarily to use the analytical method, and then the further 2D or 3D FEM is usually adopted to verify and optimize analytical results.

**Figure 2:**
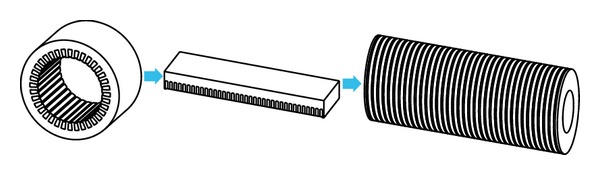
Transformation process between rotary generator and TPMLWG.

There are plenty of geometry dimensions to be determined in the generator design process, where the two main dimensions are the diameter and length of the armature iron core, respectively. The design program of TPMLWG has to calculate the mechanical structure and electromagnetic design in cylindrical coordinate, which makes the calculation process more complex and produces inaccurate analysis results. So, a new design method is presented, namely, the mathematical model of permanent magnet rotary generator in the rectangular coordinate according to the equivalent relationship between the mechanical size of permanent magnet rotary generator and the mechanical size of TPMLWG, which is established to calculate the main dimensions *D*_*i*1_ and *l*_ef_ and determine the serial number of turns *N* per phase. The conversion process is shown in Figure 2. On the basis of previous research in the domain of permanent magnet rotary generator, a large number of empirical formulas can be referred to designing the TPMLWG to adopt the new method proposed in the paper. By taking advantage of 1 kW TPMLWG, the design method is introduced.

### 2.3. Electromagnetic Design of TPMLWG

On the basis of the principle that the area of slot and axial length of the armature windings is constant, an equivalent mathematical model of TPMLWG is established according to the transformation relationship between the linear velocity of permanent magnet rotary generator and the operating speed of TPMLWG.

The mathematical model, which is used to calculate main dimensions of permanent magnet rotary generator, is shown in the following formula [14]:

(1) $$\begin{array}{c} V=D^{2}l_{\text{ef}}=\frac{6.1}{a_{p}^{\prime }K_{Nm}K_{dp}AB_{\delta }}\cdot \frac{K_{E}P_{N}}{\cos\varphi_{N}}\cdot \frac{1}{n}, \end{array}$$

where* V* is the relation coefficient of the main parameters,* D* is the armature diameter, *l*_ef_ is the effective length of armature, *a*_*p*_′ is the pole-arc coefficient, *K*_*Nm*_ is the waveform coefficient of air gap magnetic field, *K*_*dp*_ is the winding factor,* A* is the line current density, *B*_*δ*_ is the air gap flux density, *K*_*E*_ is the ratio of induced electromotive force and terminal voltage in the rated load, *P*_*N*_ is the rated power, cos⁡*φ*_*N*_ is the power factor, and *n* is the rated speed of generator.

The conversion formulas of the inside diameter of permanent magnet rotary generator* D*_*i*1_ and the axial length of TPMLWG *l*_ef-*T*_ are shown in the following formula:

(2) $$\begin{array}{c} D_{i1}=\sqrt[{3}]{\frac{2p}{\lambda \pi }V},\\ l_{\text{ef-}T}=\pi D_{i1}. \end{array}$$

In formula (2), *p* is the number of pole pairs and *λ* is the ratio of the effective length of armature *l*_ef_ and the pole pitch *τ*.

The conversion formulas of the effective length of permanent magnet rotary generator iron core *l*_ef_ and the primary outside diameter of TPMLWG *D*_*i*1-*T*_ are shown in the following formulas, respectively:

(3) $$\begin{array}{c} l_{\text{ef}}=\frac{V}{D_{i1}^{2}}, \end{array}$$

(4) $$\begin{array}{c} D_{i1\text{-}T}=\frac{l_{\text{ef}}}{\pi }+h_{0}+h_{1}+h_{2}, \end{array}$$

where *h*_0_, *h*_1_, and *h*_0_ are slot height of TPMLWG.

Yoke height *h*_*j*_ and teeth height *ht* are constant in the equivalent transformation process. The primary inside diameter of TPMLWG *D*_1-*T*_ is shown in the following formula:

(5) $$\begin{array}{c} D_{1\text{-}T}=D_{i1\text{-}T}-h_{j}-h_{t}. \end{array}$$

**Figure 3:**
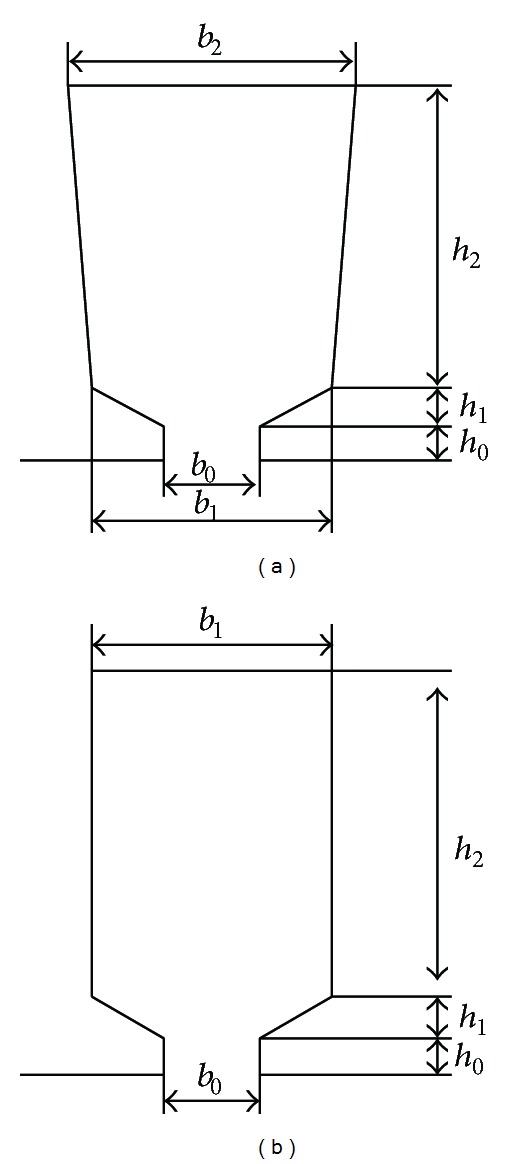
Slot shape of permanent magnet generator: (a) slot shape of permanent magnet rotation generator and (b) slot shape of TPMLWG.

In the equivalent conversion process of slot structure, the rectangular slot structure makes the adjacent stator teeth of TPMLWG parallel. The slot structure is shown in Figure 3. Rectangular slot size is transformed from designed trapezoidal slot size, and the primary teeth width should be constant.

Small power AC machine adopts a single-layer chain winding, and TPMLWG has the same induction electromotive force with permanent magnet rotary generator whose rated power is *X* kW and rated speed is *n*_1*N*_ working at low speed *n*_1_ = *n*_1*N*_/*X*. Assume that, in the design process of winding wire rules, the induction electromotive force of the armature winding is not influenced by the end effect and introduce the voltage correction coefficient and redesign the series turns of permanent magnet rotary generator perphase to further equivalent to TPMLWG.

The mathematical model of TPMLWG is established from permanent magnet rotary generator. Therefore, the major structure sizes of TPMLWG are determined. The main parameters of 1 kW TPMLWG are shown in Table 1.

### 2.4. New Secondary Structure of TPMLWG

**Figure 4:**
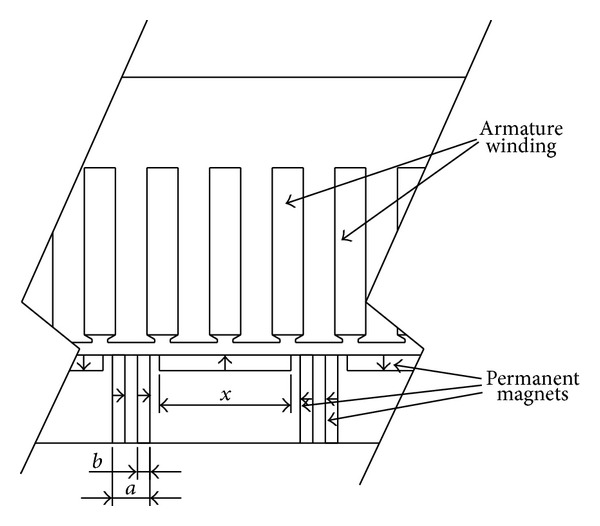
The local section of the new secondary structure.

The surface-mounted permanent magnet motors own the faster dynamic response and lower torque ripple but smaller power density. The interior permanent magnet motors have higher output power but more magnetic leakage flux. Keep the total volume of permanent magnet as a constant and divide the single traditional surface-mounted TPMLWG or the interior TPMLWG into the surface-mounted part and interior part, where the former attracts magnetic flux and decreases the leakage and the later produces the main magnetic flux. These two parts form a series-parallel hybrid magnetic circuit and a new secondary structure of the TPMLWG is established, as shown in Figure 4. This structure effectively reduces the weight of the yoke and meanwhile improves the sinusoidal characteristics of the air gap flux density by changing the structure parameters of permanent magnet* x*,* a*,* b*.

The PM structure parameters of new secondary structure are shown in Table 2.

## 3. The Simulation and Analysis Using Finite-Element Method

### 3.1. The Simulation Using Finite Element

**Figure 5:**
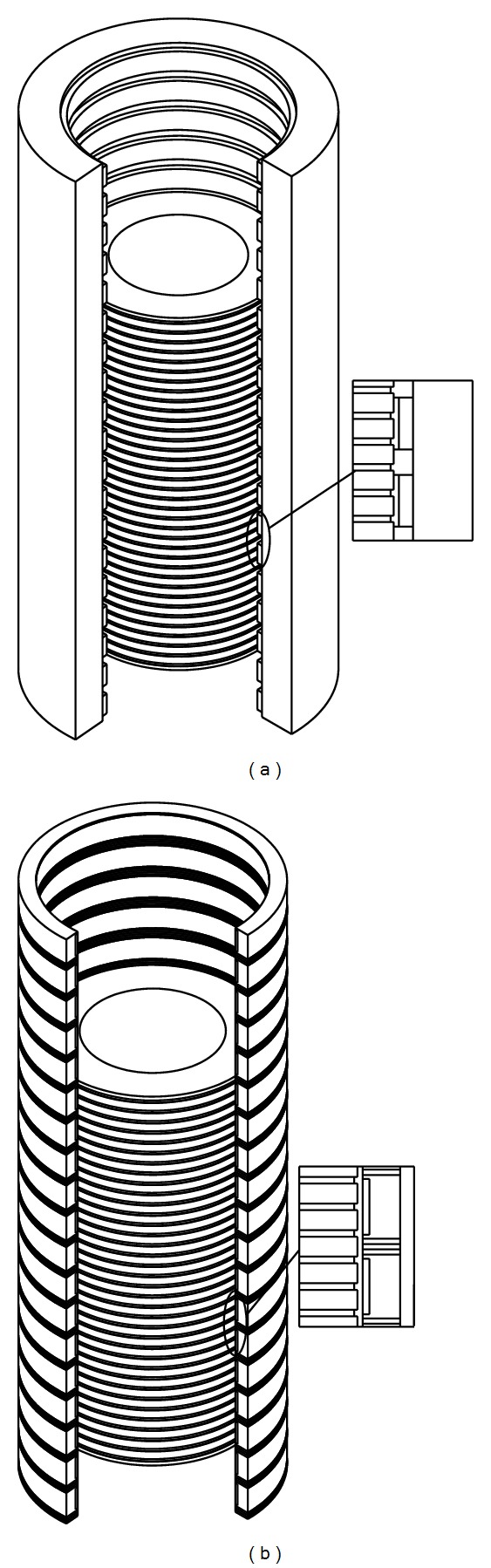
FEA model of TPMLWG: (a) the traditional surface TPMLWG and (b) the new secondary structure TPMLWG.

The finite-element models of the traditional surface-mounted TPMLWG and new secondary structure TPMLWG are shown in Figure 5.

### 3.2. The Results of Simulation and Analysis

**Figure 6:**
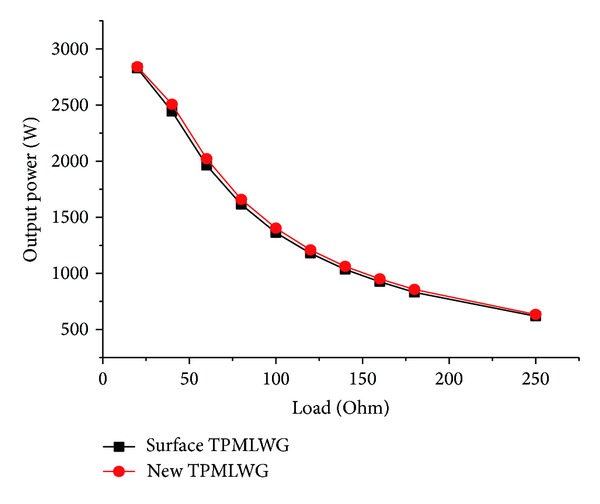
The output power of the TPMLWG in different load.

*a*_*p*_′ = 0.7, *a* = 6, and *b* = 4 are the optimum structure parameters got by comparison of the cogging force, the output power, and the sinusoidal characteristics of air gap flux density under different structure parameters of the new secondary structure TPMLWG. The traditional surface-mounted TPMLWG and the new secondary structure TPMLWG with pure resistance load are simulated. The output power of the two kinds of TPMLWG is shown in Figure 6.

The new secondary structure TPMLWG owns less magnetic flux leakage and greater air gap flux density; the output power of the new secondary structure TPMLWG is bigger, about 30 W, than the traditional surface-mounted TPMLWG at rated load.

The cogging force fluctuation cycle is 30 mm, and the skewed slot and fractional slot windings are adopted to reduce the influence of the cogging effect and then change parameters and sizes of permanent magnet to reduce the fluctuation of force [15, 16].

**Figure 7:**
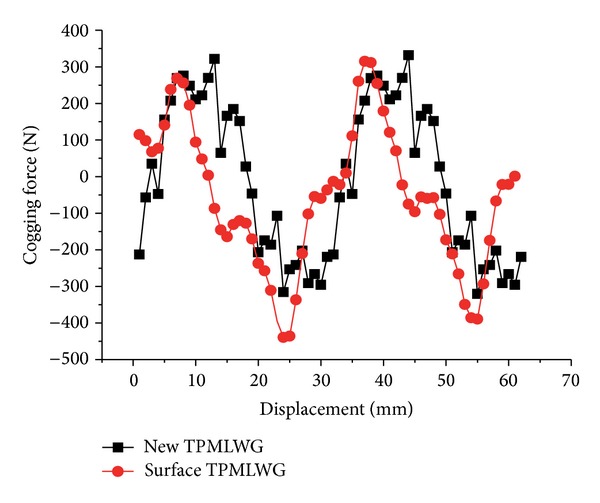
The waveform of cogging force of the traditional surface TPMLWG and the new secondary structure TPMLWG.

The detent force of TPMLWG is composed of the end-effect force and cogging force. Because of the end effect, the cogging force in Figure 7 is asymmetric. As Figure 7 shows, the maximum cogging force is *F*_*c*_ = 341.69 N under the new secondary structure and the maximum cogging force of the traditional surface-mounted TPMLWG is *F*_*c*_ = 395.97 N; the value reduced by 16%. Thus, the new secondary structure TPMLWG has smaller noise and vibration, and the stability of the TPMLWG operation also improved in the process of work.

**Figure 8:**
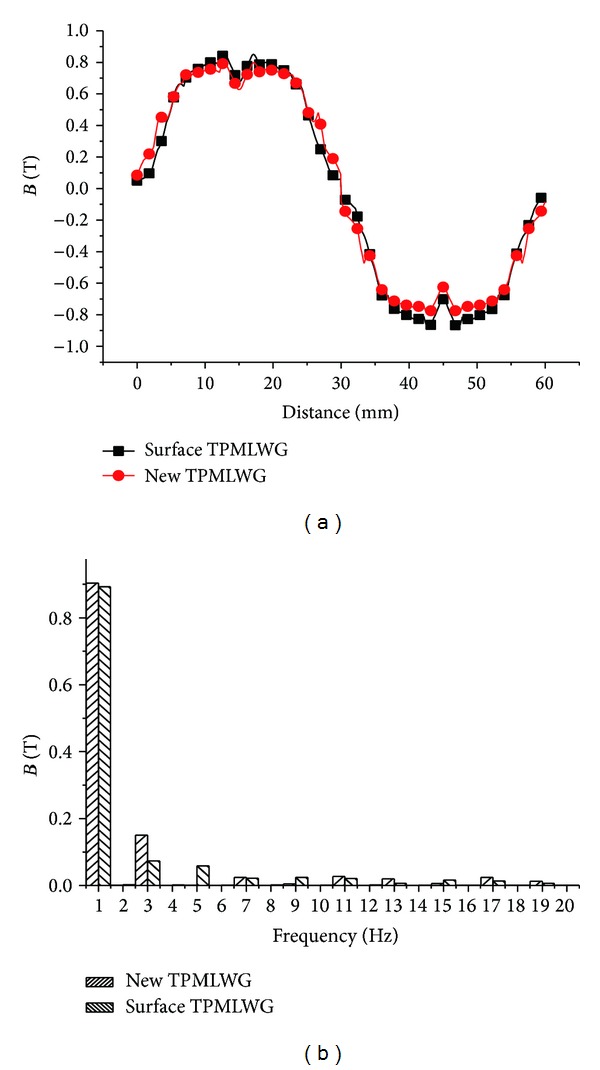
Waveform of air gap flux density and harmonic analysis: (a) the air gap flux density of TPMLWG and (b) the harmonic analysis of TPMLWG.

Under different secondary structure, the Fourier decomposition of the air gap flux density is shown in Figure 8.

Figure 8 shows that the harmonic content of air gap flux density of the new secondary structure TPMLWG is 22%, and the harmonic content of air gap flux density of the traditional surface-mounted TPMLWG is 24%; thus, the sinusoidal characteristics of the air gap flux density are improved.

Compared with the traditional surface-mounted TPMLWG, the new secondary structure TPMLWG has higher efficiency and smaller cogging force by setting reasonable structure parameters of permanent magnets; at the same time, the sinusoidal characteristics of the air gap flux density and the stability of generator operation are improved and the harmonic content is reduced effectively.

### 3.3. Analysis of Temperature Field

**Figure 9:**
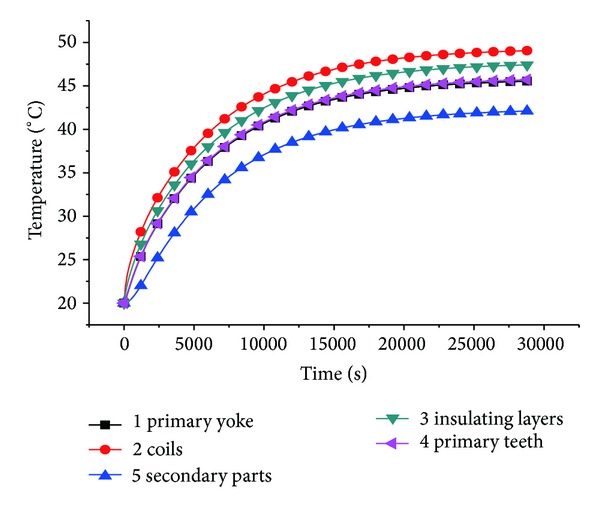
Each part temperature rising curve of the new secondary structure TPMLWG.

Adopting the finite-element analysis method, the temperature of each part of the generator is solved in rated state. The primary yoke, the armature windings copper core, the armature equivalent insulating layer, the primary teeth part, and the secondary part are selected to measure the temperature. The temperature rising curves are shown in Figure 9.

The loss is mainly copper loss of the armature windings in rated operation state [17], and it will be converted into heat energy. The stable temperature of the winding copper core is around 50°C in Figure 9, which indicates that the generator operation performance is satisfactory and meets the design requirements. The stable temperature of the secondary part is the lowest, and that is because some of the heat transferred from the primary iron core to the air gap of the TPMLWG is lost, and the rest of the heat is transferred to secondary part. In addition, the iron core loss of the secondary part is small.

**Figure 10:**
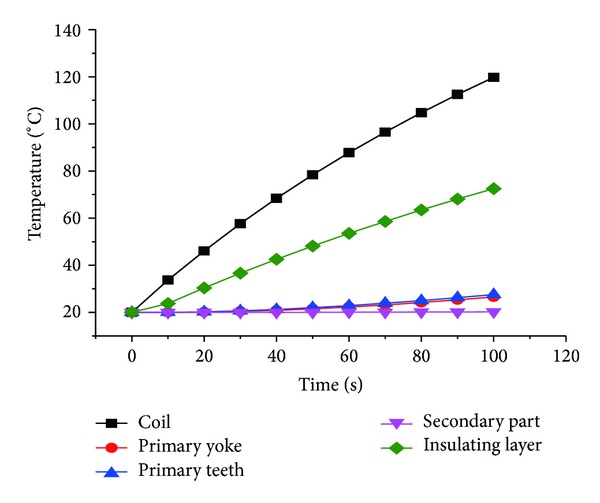
The temperature rising curve of the new secondary structure TPMLWG in three-phase short circuit.

The TPMLWG runs 110 s under the condition of three-phase short circuit, and the temperature curve of all parts is shown in Figure 10.

The winding temperature of the new secondary structure TPMLWG rises quickly under the condition of three-phase short circuit, the winding temperature reaches 120°C after operating 100 s, and the insulation class reaches the ultimate temperature of* E* class, which will expedite the insulation aging and even damage the armature windings severely.

## 4. Conclusion

The design and optimization process of TPMLWG are proposed in the paper; aiming at the design method of TPMLWG and optimization of secondary structure, the following conclusions are obtained.

A new design method is proposed in this paper. The equivalent conversion mathematical model of main dimensions of the TPMLWG is established based on the relationship between the linear velocity of the permanent magnet rotation generator and the operation speed of the TPMLWG.

A new secondary structure is presented in this paper. The secondary structure of TPMLWG contains both surface-mounted permanent magnets and interior permanent magnets, which form the series-parallel hybrid excitation magnetic circuit. Compared with the traditional secondary structure TPMLWG, the efficiency of the new secondary structure is higher, and the sinusoidal characteristic of the air gap flux density is improved, and the cogging force and the mechanical vibration are smaller during the work process.

The insulation class of the insulating material is determined by analyzing the temperature rise of each part when TPMLWG is at rated state or the three-phase short circuit state. In rated state, it is shown that the TPMLWG has low stable temperature rise and excellent operation performance. Under the three-phase short circuit state, the temperature rise of the TPMLWG reaches the ultimate temperature of* E* insulation class for short time; therefore, some protection equipment must be taken into account.

## Figures and Tables

**Table 1:** TPMLWG structure parameters.

The structure parameter	Size (unit)
The primary inner diameter	70.0 mm
The primary outer diameter	134.0 mm
The secondary outer diameter	160.0 mm
The air gap	2.0 mm
The axial length	361 mm
The number of slots	36
The number of conductors per slot	78
The nominal diameter of conductor	1.06 mm

**Table 2:** The structure parameters of permanent magnet.

The structure parameter	Size (unit)
*a*	6 mm
*b*	2 mm
*a*′_*p*_	0.7
